# Increases in *Bdnf* DNA Methylation in the Prefrontal Cortex Following Aversive Caregiving Are Reflected in Blood Tissue

**DOI:** 10.3389/fnhum.2020.594244

**Published:** 2020-11-24

**Authors:** Hannah B. D. Duffy, Tania L. Roth

**Affiliations:** Department of Psychological and Brain Sciences, University of Delaware, Newark, DE, United States

**Keywords:** *Bdnf*, blood, cross-tissue correlation, DNA methylation, early-life stress, epigenetics, maltreatment, prefrontal cortex

## Abstract

Child maltreatment not only leads to epigenetic changes, but also increases the risk of related behavioral deficits and mental disorders. These issues presumably are most closely associated with epigenetic changes in the brain, but epigenetic changes in peripheral tissues like blood are often examined instead, due to their accessibility. As such, the reliability of using the peripheral epigenome as a proxy for that of the brain is imperative. Previously, our lab has found aberrant methylation at the *Brain-derived neurotrophic factor* (*Bdnf*) gene in the prefrontal cortex of rats following aversive caregiving. The current study examined whether aversive caregiving alters *Bdnf* DNA methylation in the blood compared to the prefrontal cortex. It was revealed that DNA methylation associated with adversity increased in both tissues, but this methylation was not correlated between tissues. These findings indicate that group trends in *Bdnf* methylation between blood and the brain are comparable, but variation exists among individual subjects.

## Introduction

An aversive environment in early life can lead to numerous complications, including structural alterations to the brain and the stress response system and social and cognitive deficits (Cicchetti and Toth, 2005; De Bellis, 2005; Gould et al., 2012). Additionally, child maltreatment puts one at greater risk for numerous mental disorders, including depression, bipolar disorder, anxiety, and schizophrenia (Gibb et al., 2003; Widom et al., 2007; Li et al., 2016; Bahari-Javan et al., 2017; Kefeli et al., 2018). Epigenetic alterations such as DNA methylation are also a product of early-life adversity (Weaver et al., 2004; McGowan et al., 2009; Roth et al., 2009; Franklin et al., 2010; Unternaehrer et al., 2015). DNA methylation is an epigenetic mechanism marked by a methyl group bound to a cytosine in the DNA sequence, and it is typically associated with decreased gene expression (Moore et al., 2013). These epigenetic changes may be an origin of associated neurological and behavioral alterations.

The *Brain-derived neurotrophic factor* (*Bdnf*) gene is involved in brain development, neuroplasticity, and synaptic transmission, and changes in its gene expression have been associated with numerous mental disorders (Binder and Scharfman, 2004; Zheng et al., 2012; Zheleznyakova et al., 2016). DNA methylation at the *Bdnf* gene has been seen in both brain tissue (in animal models) and blood (in humans) following aversive caregiving or maltreatment (Roth et al., 2009, 2014; Blaze et al., 2013; Perroud et al., 2013; Thaler et al., 2014; Unternaehrer et al., 2015; Doherty et al., 2016). Such increased DNA methylation and corresponding reductions in gene expression could be leading to some of the adverse effects associated with child maltreatment.

Our lab has previously demonstrated that aversive caregiving leads to increased DNA methylation at *Bdnf* exon IX but not IV in the prefrontal cortex (PFC) of infant rodents (Roth et al., 2009; Doherty et al., 2019). The PFC is an important region to investigate as both early-life adversity and mental disorders have been linked to functional deficits in this brain region (De Bellis, 2005; Noble et al., 2005; Gould et al., 2012; Rock et al., 2014; Zhou et al., 2015). It is more difficult to observe epigenetic changes in human brains, and most often this is only possible post-mortem. Instead, many human studies observe epigenetic changes in peripheral tissues like blood. For example, increased *Bdnf* DNA methylation at exons I, IV, and VI has been seen in the blood of adults who experienced child maltreatment (Perroud et al., 2013; Thaler et al., 2014; Unternaehrer et al., 2015). However, researchers are still investigating how reliably epigenetic changes in the blood mirror that of the brain. Some studies suggest that methylation at very few CpG sites in the genome are strongly correlated between blood and the brain (Hannon et al., 2015; Walton et al., 2016; Braun et al., 2019). Others have found correlations in global and gene-specific methylation between blood and brain as well as similar methylation patterns between the two tissues (Ursini et al., 2011; Horvath et al., 2012; Ewald et al., 2014; Kundakovic et al., 2015).

The current study aimed to investigate how closely *Bdnf* methylation patterns in the blood reflect that of the PFC following aversive caregiving. DNA methylation within both of these tissues was examined in infant pups following a scarcity-adversity rodent model of low nesting resources. Because early-life adversity has previously been shown to alter methylation at these exons (Roth et al., 2009; Kundakovic et al., 2015; Doherty et al., 2019) and because their corresponding transcripts appear highly expressed in blood (Aid et al., 2007; Cattaneo et al., 2016), *Bdnf* exons IV and IX were selected for initial investigation. Additionally, methylation correlations between the two tissues were investigated. This research holds valuable insights into the reliability of drawing conclusions about the brain from the epigenome of peripheral tissue.

## Materials and Methods

### Subjects

Male and female Long-Evans pups were bred in-house at the University of Delaware. First-born litters were not used to ensure that all dams were experienced mothers prior to experimentation. The date of birth was designated postnatal day (PN) 0, and litters were culled to 5–6 pups of each sex on PN1. Animals were given food and water *ad libitum*. They were housed in polypropylene cages with ample bedding in a temperature- and light-controlled room. A 12-h light-dark cycle (07:00–19:00) was employed, with all behavioral manipulations performed during the light cycle. All practices were in accordance with and approved by the University of Delaware Animal Care and Use Committee.

### A Rodent Model for Aversive Caregiving

The scarcity-adversity model of low nesting resources employed in this study has been previously described and shown to induce more aversive maternal behaviors compared to control conditions (Roth et al., 2009; Blaze et al., 2013; Doherty et al., 2016, 2019). Pups in each litter were randomly split into thirds and placed in either the normal maternal care, cross-foster care, or maltreatment condition. For 30 min each day from PN1 through 7, cross-foster care and maltreatment pups were placed with another dam while the normal care pups remained with their biological dam, only being removed from the home cage to be marked for identification and weighed each day. These other dams were lactating, fed the same diet, and postpartum age-matched to the experimental pups, so as not to impact caregiving (Purcell et al., 2011; Grieb et al., 2018). Data suggest that pups cannot distinguish between their biological mother and age-matched dams fed the same diet (Leon, 1975). The cross-foster care pups were placed with a dam who was given an hour to habituate to a plexiglass chamber with plenty of nesting materials. The pups in the maltreatment condition were placed with a dam with no time to habituate to a plexiglass chamber and inadequate nesting materials, conditions previously shown to elicit aversive behaviors (Roth et al., 2009; Blaze et al., 2013; Doherty et al., 2016, 2019). Video cameras recorded each 30-min session, and a trained observer scored a random subset (*n* = 7 litters) for presence of nurturing (licking and grooming, hovering, and nursing) and aversive (dropping, dragging, roughly handling, actively avoiding, and stepping) behaviors in 5-min bins.

### Tissue Collection

Brain and blood extractions were performed on PN8. A subset of animals underwent brain perfusions via a saline (0.9% NaCl) flush to ensure that no blood in the PFC tissue was confounding results. These animals were anesthetized with ketamine (80 mg/kg) and xylazine (10 mg/kg), and whole blood was extracted directly from the left ventricle prior to perfusions. In the remaining animals, that were not perfused, whole blood was collected at the neck following decapitation. All blood tissue was collected in tubes containing 50 μl EDTA to prevent coagulation. PFC tissue was extracted and homogenized. Whole blood and PFC were stored in −20°C and −80°C freezers, respectively.

### DNA Methylation

DNA was extracted from blood and the PFC and bisulfite converted (Qiagen Inc.). Methylation-specific PCR (MSP) was performed with methylation-specific primers designed for *Bdnf* exons IV and IX (Roth et al., 2009). The comparative Ct method was employed to establish fold change in DNA methylation compared to normal care controls, using tubulin as a reference gene (Livak and Schmittgen, 2001; Pfaffl, 2001).

### Statistical Analysis

A two-way ANOVA (infant condition x behavior type) with Sidak's *post-hoc* test was used to analyze maternal behavior. A one-way ANOVA with Tukey's *post-hoc* test was used to analyze the ratio of aversive to total maternal behaviors across the three conditions. Two-way ANOVAs were used to analyze DNA methylation results with the factors being either perfusion (perfusion or no perfusion) and infant condition (cross-foster or maltreatment) or sex and infant condition. After collapsing across sex and perfusion, unpaired two-sample *t*-tests or Mann-Whitney tests (if there was no homogeneity of variance) were used to compare DNA methylation between maltreatment and cross-foster conditions. One-sample *t*-tests were used to compare DNA methylation in cross-foster and maltreatment groups to a theoretical normal care mean of one. Linear regressions of fold change in DNA methylation were run between the PFC and blood. If there was more than one subject in any group from the same litter, they were averaged to avoid within-litter effects. Significance was set to *p* < 0.05.

## Results

### Maternal Behavior

**Figure 1:**
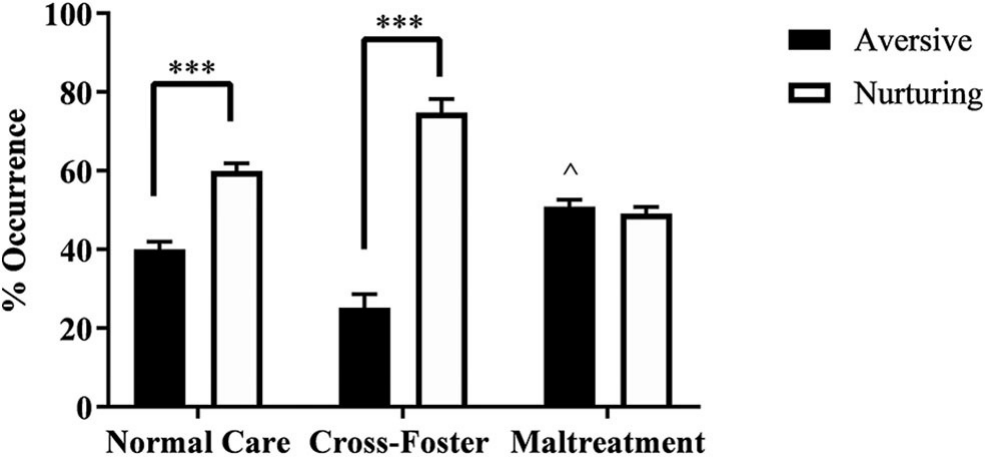
Percent occurrence of aversive and nurturing behaviors across the three infant conditions (normal care, cross-foster care, and maltreatment). ***p < 0.001, ^∧^*p* < 0.05 compared to normal and cross-foster care; error bars represent standard error of the mean.

Pups were randomly placed into either the normal maternal care, cross-foster care, or maltreatment condition. A two-way ANOVA examining behavior type (nurturing or aversive) and infant condition (normal care, cross-foster, or maltreatment) revealed a main effect of behavior type [*F*_(1, 34)_ = 117.6, *p* < 0.001] and an interaction [*F*_(2, 34)_ = 50.41, *p* < 0.001]. *Post-hoc* analyses indicated that both normal care (*p* < 0.001) and cross-foster care (*p* < 0.001) conditions had more nurturing behaviors compared to aversive behaviors, while there was no significant difference between nurturing and aversive behaviors in the maltreatment condition (Figure 1). A one-way ANOVA examining the aversive behaviors to total maternal behaviors ratio revealed a main effect of infant condition [*F*_(2, 17)_ = 25.21, *p* < 0.001]. *Post-hoc* analyses revealed a significantly higher ratio in the maltreatment condition compared to normal care (*p* = 0.022) and cross-foster care (*p* < 0.001) conditions. Additionally, the cross-foster care condition had a significantly lower ratio compared to normal care controls (*p* = 0.002), an unexpected result; cross-foster and normal care groups typically do not display differences in behavior (Blaze et al., 2013; Roth et al., 2014).

### Perfusions

Fold change in DNA methylation compared to normal care controls was calculated for both maltreatment and cross-foster care animals. In two-way ANOVAs (perfusion x infant condition), no main effect of perfusion was found in blood or the PFC at exon IV or IX in males or females (Supplementary Table 1). As a result, perfusion and no perfusion groups were collapsed for the remaining analyses.

### DNA Methylation Increases in Maltreated Animals

**Figure 2:**
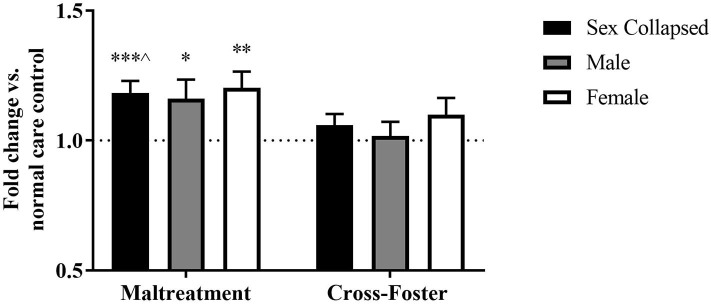
Fold change in *Bdnf* DNA methylation at exon IV in the PFC in the maltreatment and cross-foster groups graphed vs. normal maternal care (dotted line). *n* = 12–21 per group; *p < 0.05 vs. normal maternal care (NMC); **p < 0.01 vs. NMC; ***p < 0.001 vs. NMC; ^∧^*p* < 0.1 vs. cross-foster care; error bars represent standard error of the mean.

Changes in DNA methylation following aversive caregiving were investigated in both blood and the PFC at exon IV. In the PFC, a two-way ANOVA revealed no main effect of sex or an interaction but a trending effect of infant condition [*F*_(1, 124)_ = 3.750, *p* = 0.055]. One-sample *t*-tests revealed a significant increase in maltreatment males [*t*_(31)_ = 2.204, *p* = 0.035] and females [*t*_(31)_ = 3.287, *p* = 0.003] compared to normal care controls (Figure 2). When collapsing by sex, the maltreatment group was again significantly above normal care controls [*t*_(63)_ = 3.829, *p* < 0.001] and trending above cross-foster care animals [*t*_(126)_ = 1.924, *p* = 0.057].

**Figure 3:**
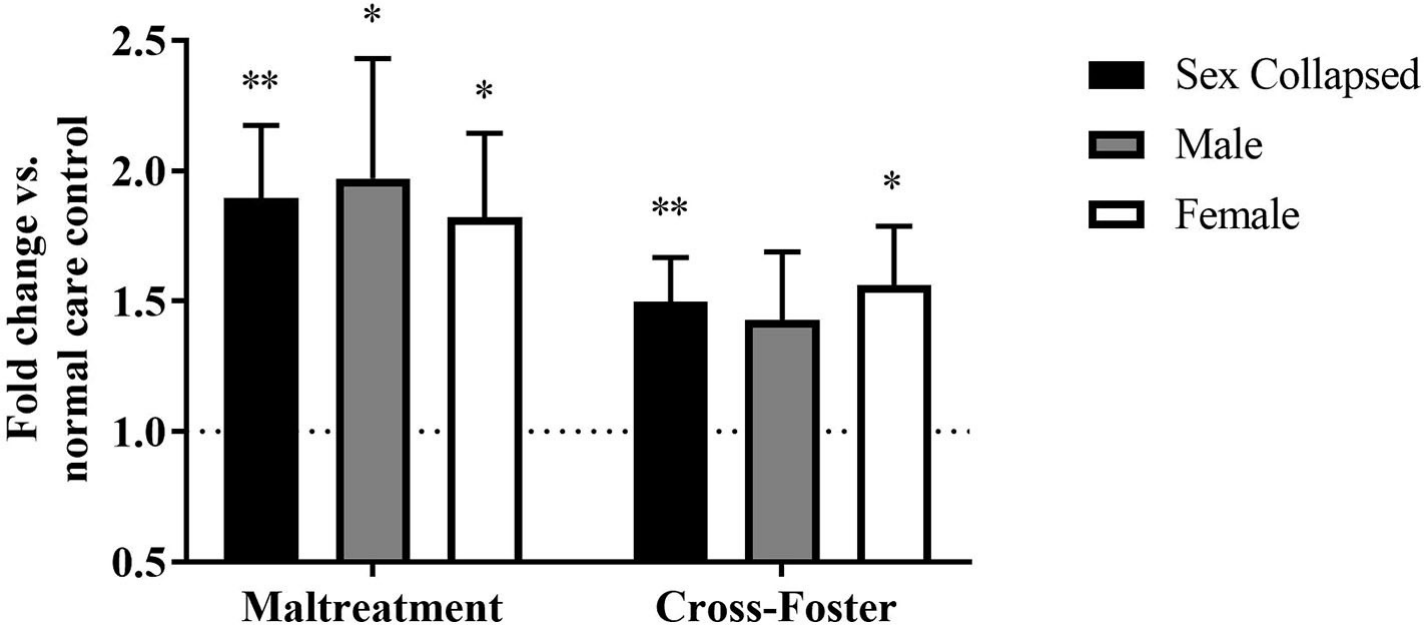
Fold change in *Bdnf* DNA methylation at exon IV in blood in the maltreatment and cross-foster groups graphed vs. normal maternal care (dotted line). *n* = 12–21 per group; *p < 0.05 vs. normal maternal care (NMC); **p < 0.01 vs. NMC; error bars represent standard error of the mean.

The DNA methylation in whole blood tissue was similar to that of the PFC at exon IV. A two-way ANOVA revealed no main effect of sex, infant condition, or an interaction. However, one-sample *t*-tests showed that maltreatment males [*t*_(31)_ = 2.111, *p* = 0.043] and females [*t*_(31)_ = 2.532, *p* = 0.017] exhibited significantly more methylation than normal care controls (Figure 3). Once sexes were collapsed, the maltreatment group was again significantly increased compared to normal care controls [*t*_(63)_ = 3.207, *p* = 0.002] but not cross-foster care animals.

**Figure 4:**
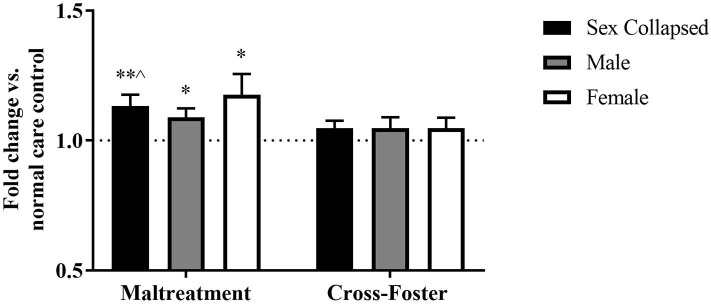
Fold change in *Bdnf* DNA methylation at exon IX in the PFC in the maltreatment and cross-foster groups graphed vs. normal maternal care (dotted line). *n* = 12–21 per group; *p < 0.05 vs. normal maternal care (NMC); **p < 0.01 vs. NMC; ^∧^*p* < 0.1 vs. cross-foster care; error bars represent standard error of the mean.

Methylation changes due to aversive caregiving were also examined at exon IX. In the PFC, a two-way ANOVA revealed no main effect of sex, infant condition, or an interaction, but maltreatment males [*t*_(31)_ = 2.635, *p* = 0.013] and females [*t*_(31)_ = 2.188, *p* = 0.036] both exhibited significantly more methylation than normal care controls (Figure 4). With sexes collapsed, the maltreatment group was significantly increased compared to normal care controls [*t*_(63)_ = 3.044, *p* = 0.003] and trending above the cross-foster care group (U = 1,665, *p* = 0.068).

**Figure 5:**
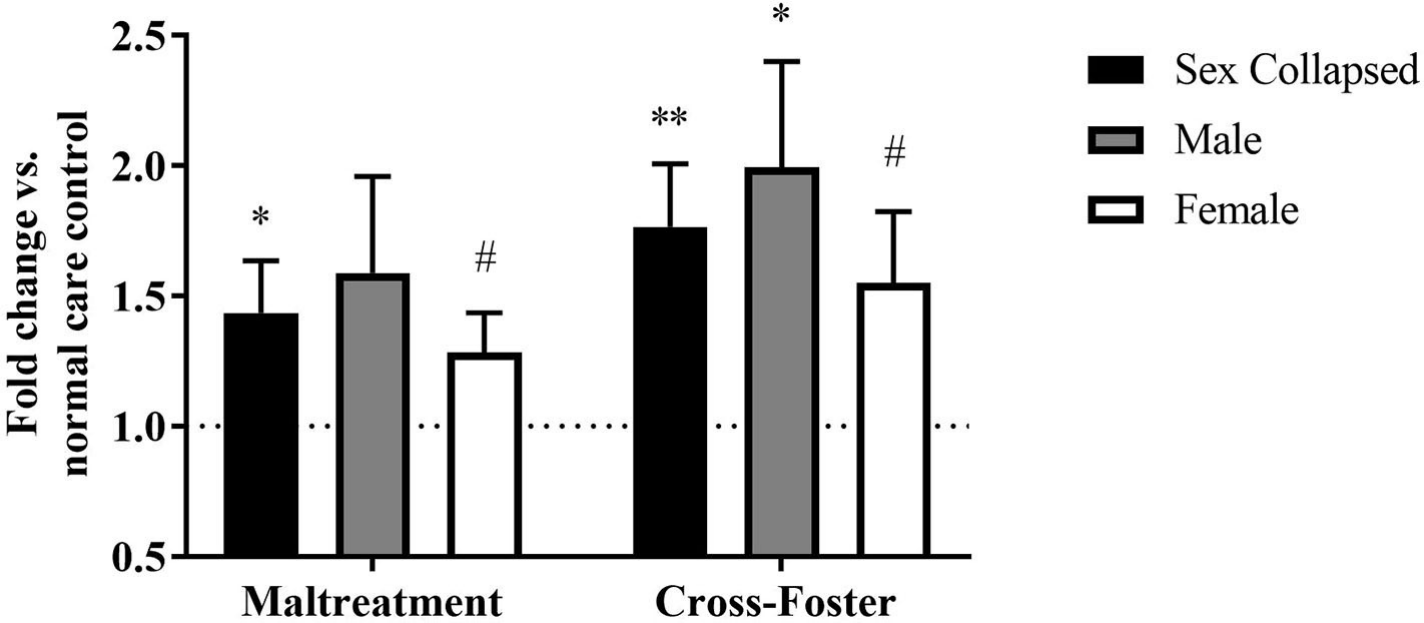
Fold change in *Bdnf* DNA methylation at exon IX in blood in the maltreatment and cross-foster groups graphed vs. normal maternal care (dotted line). *n* = 12–21 per group; *p < 0.05 vs. normal maternal care (NMC); **p < 0.01 vs. NMC; ^#^*p* < 0.1 vs. NMC; error bars represent standard error of the mean.

Similar methylation changes were seen in the blood at exon IX (Figure 5). A two-way ANOVA again revealed no main effect of sex, infant condition, or an interaction. Maltreatment females were trending above normal care controls [*t*_(31)_ = 1.879, *p* = 0.070], but maltreatment males were not significantly different. However, after collapsing across sex, the maltreatment group was significantly increased compared to normal care controls [*t*_(63)_ = 2.191, *p* = 0.032], although not compared to cross-foster animals.

### DNA Methylation Increases in Cross-Foster Care Animals' Blood

Fold change in DNA methylation in the cross-foster group was also compared to the normal care condition. No significant difference was found between cross-foster care animals and normal care controls in the PFC (Figures 2, 4). However, in the blood at exon IV, significant increases compared to normal care controls were present in cross-foster females [*t*_(32)_ = 2.507, *p* = 0.017] and cross-foster (sex collapsed) [*t*_(63)_ = 2.934, *p* = 0.005] (Figure 3). At exon IX in blood, cross-foster females were trending above normal care controls [*t*_(32)_ = 2.016, *p* = 0.052] while males [*t*_(30)_ = 2.445, *p* = 0.021] and the sex collapsed group [*t*_(63)_ = 3.166, *p* = 0.002] displayed significantly increased methylation compared to normal care (Figure 5).

### Correlations in DNA Methylation Between Blood and the PFC

**Figure 6:**
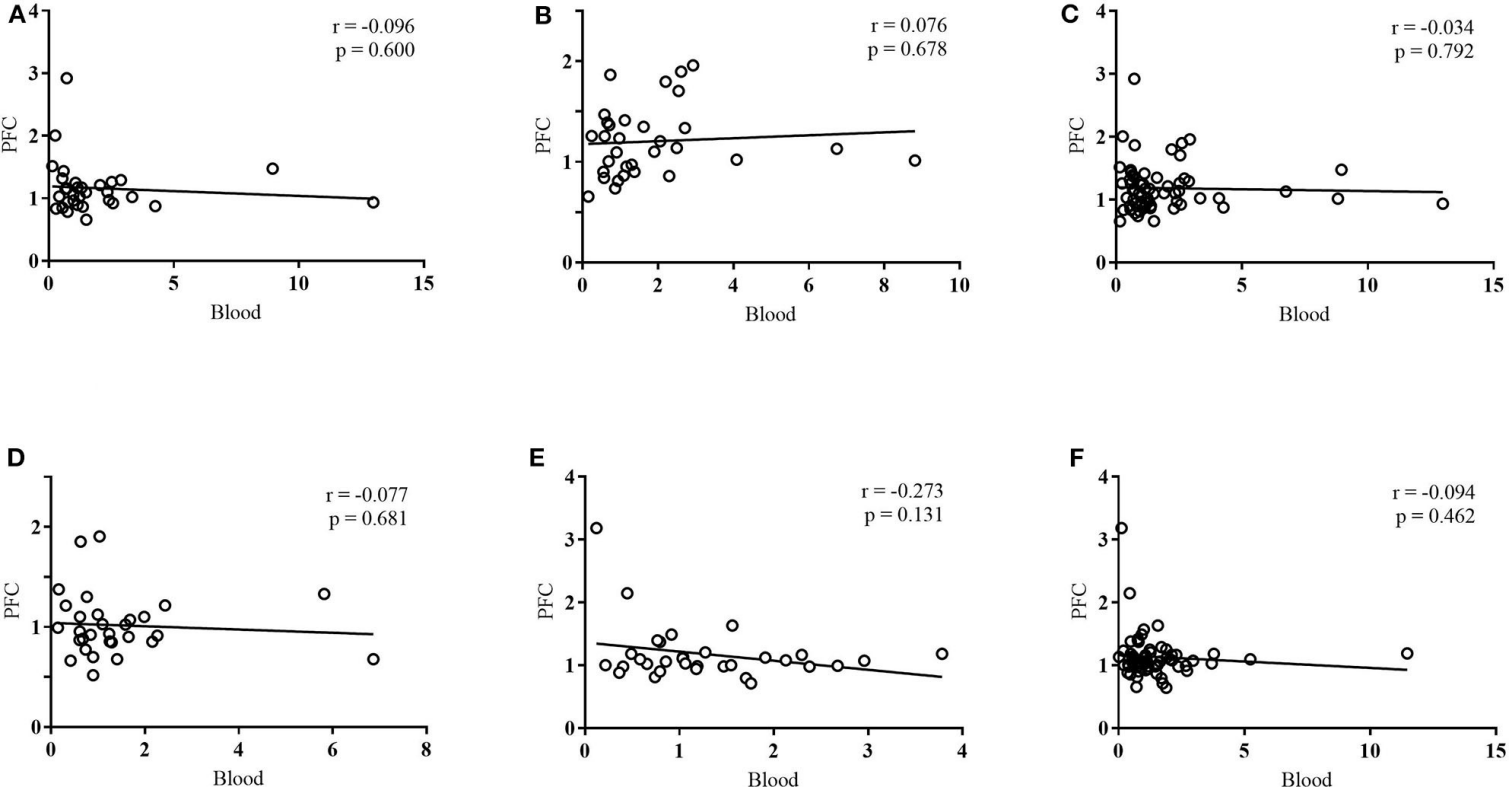
Linear regressions between PFC and blood DNA methylation at **(A)** exon IV in maltreatment males, **(B)** exon IV in maltreatment females, **(C)** exon IV in maltreatment: sex collapsed, **(D)** exon IV in cross-foster males, **(E)** exon IX in maltreatment females, **(F)** exon IX in maltreatment: sex collapsed. DNA methylation was quantified in terms of fold change vs. normal care controls. *n* = 12–21 per group.

Linear regressions were run for any group that exhibited the same directional change in methylation in both blood and the PFC (an increase, decrease, or no change in both tissues) (Figure 6). At exon IV, this included maltreatment males, maltreatment females, maltreatment (sex collapsed), and cross-foster males. At exon IX, this included maltreatment females and maltreatment (sex collapsed). No significant correlations were found between blood and the PFC for any of these groups. Using a method based on that of Davies et al. (2012), mean difference scores of the log transformed data were analyzed, but also failed to reveal any significant correlations.

## Discussion

Since there was no main effect of perfusion in either sex, either exon, or either tissue, it can be concluded that the remaining blood in the rodent brains was not confounding brain methylation results. The blood was presumably in too small a quantity to alter methylation quantification. This suggests future studies exploring methylation in this tissue can gain an accurate reading of brain methylation without performing perfusions.

Once collapsing the data by perfusion, an increase in DNA methylation at exon IV in the maltreatment group compared to normal care controls was found in both tissues. Previously, studies have found no methylation change at exon IV in the infant PFC following this aversive caregiving paradigm (Roth et al., 2009; Doherty et al., 2019). The unexpected increase at exon IV in the current study could be due to the animals being obtained from a vendor not previously used by our lab. Additionally, the sample sizes are larger in this study compared to these previous studies (Roth et al., 2009; Doherty et al., 2019). Although not previously seen in infancy, this paradigm has demonstrated an increase in exon IV methylation in the adult PFC (Roth et al., 2009). It is possible that a minor increase in methylation in infancy becomes a more prominent, and thus more visible, increase in adulthood. Additionally, the human literature has found that exon IV mRNA expression in the PFC does change over the course of early life (Wong et al., 2009), indicating that environmental factors like aversive caregiving may indeed be able to increase exon IV methylation in the PFC at this age.

Aversive caregiving also led to an increase in DNA methylation at exon IV in blood. This is in accordance with previous human studies that have found increased exon IV methylation in the blood of adults maltreated in childhood (Perroud et al., 2013; Thaler et al., 2014). To the best of our knowledge, no studies have investigated such methylation changes in infancy. The current findings suggest that this increased exon IV methylation may occur early in the lifespan.

Aversive caregiving led to increased DNA methylation at exon IV in both tissues, suggesting that methylation alterations within the two tissues may occur in tandem, at least to some extent. This affirms previous research that has found that a rodent model for early-life stress leads to increased exon IV methylation at CpG site 4 in males in the hippocampus and in blood (Kundakovic et al., 2015), along with other studies that have found significant correlations between blood and frontal cortex methylation (Ursini et al., 2011; Horvath et al., 2012). This indicates that blood is an appropriate proxy for the epigenome of the brain in some cases.

After collapsing by perfusion and sex, the maltreatment group exhibited significantly more methylation at exon IX in both the PFC and blood. This effect in the PFC was an expected replication of previous findings (Roth et al., 2009; Doherty et al., 2019). To the best of our knowledge, the increase at exon IX in blood brought on by aversive caregiving is a novel finding. However, human studies have previously found child maltreatment to be associated with hypermethylation in blood at exons I, IV, and VI (Perroud et al., 2013; Thaler et al., 2014; Unternaehrer et al., 2015). Additionally, research has suggested that methylation at exon IX in blood is susceptible to modifications (Hsieh et al., 2019). Just as it did at exon IV, DNA methylation at exon IX appears to follow the same pattern in both the PFC and blood after aversive caregiving. This has important implications for human literature that may be able to rely on using certain methylation changes in the blood as an indication of changes in the brain.

Compared to normal care controls, cross-foster animals exhibited no change in methylation at either exon in the PFC. This is in accordance with previous findings (Roth et al., 2009; Doherty et al., 2019). However, the cross-foster group did show increased DNA methylation at exons IV and IX in the blood. Some shared factor between maltreatment and cross-foster conditions, such as exposure to a novel environment or increased handling, may increase blood *Bdnf* methylation. As such, a cause and effect relationship between aversive caregiving and increased methylation in blood is not explicitly demonstrated. However, as previous literature suggests that blood *Bdnf* methylation increases following early-life adversity (Perroud et al., 2013; Thaler et al., 2014; Kundakovic et al., 2015; Unternaehrer et al., 2015), it is likely that the increase in methylation seen in the maltreatment group is, at least partially, a result of the aversive environment.

Additionally, as gene expression was not explored in this study, it is unknown how the increased DNA methylation in the blood of cross-foster care animals would impact gene expression, if at all. Previously, increased PFC methylation associated with this paradigm has been shown to correspond to decreased gene expression (Roth et al., 2009). However, DNA methylation can also be linked to increased gene expression (Blaze and Roth, 2013). If this is the case in the cross-foster care group, a similar increase in methylation in both conditions could lead to opposite effects on gene expression. Future studies should examine mRNA levels to assess how methylation changes in both cross-foster and maltreatment groups may be impacting gene expression.

No correlations were found in DNA methylation between blood and the PFC at either exon. This suggests that there is no clear-cut correlation in *Bdnf* DNA methylation between the two tissues following aversive caregiving. Despite observing the same group trends in DNA methylation between PFC and blood, there does not appear to be uniformity between tissues at the individual subject level. Additionally, if correlations between the two tissues had been significant, conclusions would have been limited as only select groups exhibited the same directional change in both tissues.

The current study used a method of methylation quantification that allows for methylation to be assessed at individual exons, but not at individual CpG sites within those exons. Previous reports that have found significant correlations between the two tissues have analyzed methylation at individual CpG sites (Ursini et al., 2011; Horvath et al., 2012; Witzmann et al., 2012; Ewald et al., 2014; Hannon et al., 2015; Kundakovic et al., 2015; Walton et al., 2016; Braun et al., 2019). Quantifying methylation at this level in the future may elucidate site-specific correlations that are otherwise impossible to detect. This is quite likely, as only select CpG sites within the genome appear strongly correlated between blood and the brain (Hannon et al., 2015; Walton et al., 2016; Braun et al., 2019). Additionally, correlations between the two tissues have been found when comparing different CpG sites in each tissue (Ewald et al., 2014), another result that would have been impossible to detect using the method employed in the current study. In the future, examining methylation at individual CpG sites would be valuable in further understanding how these tissues may be correlated.

In addition to exploring methylation at individual CpG sites, future studies may sort cells before assessing methylation changes. As both PFC and blood tissue are made up of multiple cell types, some cell populations may be more closely correlated with the opposing tissue than others. Additionally, blood cell proportions can change due to stress (Dominguez-Gerpe and Rey-Mendez, 2001). Cell sorting would allow us to detect any such proportional changes and the impact this may have on methylation levels, as different blood cells are known to exhibit varying degrees of methylation (Jaffe and Irizarry, 2014). Thus, apparent changes in blood methylation following aversive caregiving may be two-fold: a result of increased methylation in certain blood cells and a result of increased quantities of blood cells that naturally have greater methylation than others.

This study compared PFC and blood methylation at one gene impacted by early-life adversity, but future studies should explore other genes. Studies could also compare blood methylation to other brain regions, as methylation differences have been found between different regions of the brain (Roth et al., 2014). Some research suggests that methylation in saliva may bare a closer resemblance to the brain compared to blood (Smith et al., 2015; Braun et al., 2019). Future studies could explore methylation in saliva to see if this is the case after aversive caregiving. Further research could also explore methylation changes in adult rodents. After aversive caregiving, adult rodent brains have some methylation markers that were present in infancy and some novel methylation markers that arise later in life (Roth et al., 2009, 2014). This may also be the case in the blood. Knowing how blood and brain methylation compare throughout the lifespan would be critical when using blood as a proxy for the epigenome of the brain. The experimental design currently employed does not allow for an analysis of the degree to which individual animals' methylation values correlate to their caregiving experiences. Future studies should also adjust the experimental design so that this analysis can be performed.

Overall, this study found that aversive caregiving alters blood and brain DNA methylation at *Bdnf* exons IV and IX in a similar vein at the group, but not necessarily the individual, level. No correlations were found in methylation between the two tissues. Although further research is needed to more thoroughly understand the relationship between epigenetic changes in these two tissues, there is potential for blood to serve as a proxy for epigenetic changes of the brain following aversive caregiving.

## Data Availability Statement

The raw data supporting the conclusions of this article will be made available by the authors, without undue reservation.

## Ethics Statement

The animal study was reviewed and approved by University of Delaware Animal Care and Use Committee.

## Author Contributions

HBDD and TLR designed the study and took part in interpretation of the results. Animal generation, tissue collection, and biochemical and data analyses were performed by HBDD and overseen by TLR. HBDD wrote the first draft of the manuscript. TLR edited the manuscript. Both authors contributed to the article and approved the submitted version.

## Conflict of Interest

The authors declare that the research was conducted in the absence of any commercial or financial relationships that could be construed as a potential conflict of interest.
