# Supplementary material for: Increases in Bdnf DNA Methylation in the Prefrontal Cortex Following Aversive Caregiving Are Reflected in Blood Tissue
Source: Front Hum Neurosci. 2020 Nov 24;14:594244. doi: 10.3389/fnhum.2020.594244 (PMC7721665; doi:10.3389/fnhum.2020.594244)
Supplement: Supplementary file 1 [file Table_1.DOCX]

Supplementary Material

**Supplementary Table 1.** Results of two-way ANOVAs examining perfusion and infant condition at both exons (IV and IX), in both tissues (PFC and blood), and in both sexes (males and females). #p<0.1. No main effect of perfusion was found in any of these tests, so perfusion and no perfusion groups were collapsed in the remaining analyses.

|  | | | | F | DFn | DFd | p |
| --- | --- | --- | --- | --- | --- | --- | --- |
| Exon IV | PFC | Males | Perfusion | 0.578 | 1 | 59 | 0.450 |
|  |  |  | Infant Condition | 2.913 | 1 | 59 | 0.093^#^ |
|  |  |  | Interaction | 0.594 | 1 | 59 | 0.444 |
|  |  | Females | Perfusion | 1.561 | 1 | 61 | 0.216 |
|  |  |  | Infant Condition | 0.771 | 1 | 61 | 0.384 |
|  |  |  | Interaction | 1.012 | 1 | 61 | 0.318 |
|  | Blood | Males | Perfusion | 1.963 | 1 | 59 | 0.166 |
|  |  |  | Infant Condition | 0.415 | 1 | 59 | 0.522 |
|  |  |  | Interaction | 2.073 | 1 | 59 | 0.155 |
|  |  | Females | Perfusion | 0.035 | 1 | 61 | 0.852 |
|  |  |  | Infant Condition | 0.779 | 1 | 61 | 0.381 |
|  |  |  | Interaction | 0.159 | 1 | 61 | 0.691 |
| Exon IX | PFC | Males | Perfusion | 0.503 | 1 | 59 | 0.481 |
|  |  |  | Infant Condition | 0.422 | 1 | 59 | 0.518 |
|  |  |  | Interaction | 0.237 | 1 | 59 | 0.628 |
|  |  | Females | Perfusion | 0.049 | 1 | 61 | 0.826 |
|  |  |  | Infant Condition | 2.612 | 1 | 61 | 0.111 |
|  |  |  | Interaction | 0.838 | 1 | 61 | 0.364 |
|  | Blood | Males | Perfusion | 1.015 | 1 | 59 | 0.318 |
|  |  |  | Infant Condition | 0.126 | 1 | 59 | 0.723 |
|  |  |  | Interaction | 2.250 | 1 | 59 | 0.139 |
|  |  | Females | Perfusion | 0.739 | 1 | 61 | 0.394 |
|  |  |  | Infant Condition | 1.082 | 1 | 61 | 0.302 |
|  |  |  | Interaction | 0.704 | 1 | 61 | 0.405 |
